# A nomogram incorporating Ki67 to predict survival of acral melanoma

**DOI:** 10.1007/s00432-023-05127-w

**Published:** 2023-07-20

**Authors:** Yu Du, Caili Li, Lili Mao, Xiaoting Wei, Xue Bai, Zhihong Chi, Chuanliang Cui, Xinan Sheng, Bin Lian, Bixia Tang, Xuan Wang, Xieqiao Yan, Siming Li, Li Zhou, Jun Guo, Lu Si

**Affiliations:** Key Laboratory of Carcinogenesis and Translational Research (Ministry of Education, Beijing), Department of Renal Cancer and Melanoma, Peking University Cancer Hospital and Institute, No. 52 Fucheng Road, Haidian District, Beijing, 100142 China

**Keywords:** Ki67, Acral melanoma, Nomogram, Prognosis

## Abstract

**Background:**

The proliferation marker Ki67 is associated with the progression and prognosis of melanoma. However, its prognostic impact on acral melanoma (AM) remains unclear.

**Methods:**

A total of 314 AM patients were enrolled from a cohort of 5758 patients with melanoma at Peking University Cancer Hospital between 2006 and 2018. The patients were divided into Ki67 high- and low-expressing groups using a cut-off value of 30%. The associations between Ki67 and clinicopathologic characteristics as well as survival were analyzed. Cox proportional regression analysis was used to establish a nomogram to predict the survival probabilities of AM.

**Results:**

Among 314 patients, the Ki67-high group (Ki67 ≥ 30%) included 49.4% of patients at diagnosis. Patients in the Ki67-high group had lower median melanoma-specific survival (MSS) than those in the Ki67-low group (60.7 months vs. not reached, p < 0.001). In multivariate analyses, Ki67, lymph node metastasis and primary site were independent prognostic factors for MSS. The nomogram showed that Ki67 had the fourth greatest impact on survival, following Breslow thickness, lymph node metastasis and primary site. The C-index of the nomogram was 0.765 and 0.758 in the training and validation cohort, respectively. Area under the curve values were both near 0.8 in the training and validation cohorts. Net reclassification improvement and integrated discrimination improvement demonstrated that the predictive nomogram performed better than the traditional AJCC staging system.

**Conclusion:**

Ki67 expression is an independent prognostic factor for MSS in AM. A predictive model incorporating Ki67 and clinical factors was constructed to predict the prognosis of AM.

**Supplementary Information:**

The online version contains supplementary material available at 10.1007/s00432-023-05127-w.

## Introduction

Acral melanoma (AM), arising on the non-hair-bearing skin of the palms, soles or subungual regions, is a rare melanoma type in Western populations (Markovic et al. 2007). However, it is the most common subtype in Asian, African and Hispanic populations, constituting up to 40–75% of color populations (Chen et al. 2020; Desai et al. 2017). Compared with cutaneous melanoma from other sites, AM is associated with worse prognosis and poorer survival because of delayed diagnosis (Carrera and Puig-Butille 2018; Desai et al. 2017; Teramoto et al. 2018). Genomically, AM shows lower mutational burdens, different oncogenic drivers and a higher number of structural chromosomal changes (Chen et al. 2020; Shi et al. 2022). While immunotherapies and target therapies have significantly improved the survival of patients with cutaneous melanoma, these therapies are less effective or are ineffective for most patients with AM. Many risk factors, including age, Breslow thickness, ulceration status, stage, primary site, distant metastasis and lactate dehydrogenase (LDH) level have been shown to be correlated with prognosis in AM patients (Teramoto et al. 2018; Wang et al. 2021; Wei et al. 2020; Wei et al. 2022a,b). However, these results are controversial for some variables. An analysis of 853 AM patients in China depicted the prognostic value of Breslow thickness in AM, but no association was found between tumor thickness and survival in patients with Breslow thickness > 2 mm (Wei et al. 2022a). Interestingly, ulceration was found to have varying impact on prognosis across Breslow thickness. For patients with Breslow thickness ≤ 1 mm, ulceration was significantly associated with prognosis, while for patients with Breslow thickness > 1 mm, no correlation was found (Wei et al. 2022b). Wei et al. discovered that sole melanoma had worse survival compared with other subtypes (Wei et al. 2020), but subungual melanoma was considered to have worse survival than palm or sole melanoma in a previous study in Japan (Nakamura et al. 2020). The American Joint Committee on Cancer (AJCC) staging system for tumor-node-metastasis (TNM) remains the primary tool for prognostic prediction, but it has limitations because patients at the same stage can have vastly different survival outcomes (Gershenwald et al. 2017). Therefore, it is crucial to identify additional prognostic predictors and construct new predictive models for AM.

The Ki67 protein is an indicator of proliferative activity expressed in all phases of the cell cycle except G0. It has been extensively evaluated in various malignant tumor types, especially in breast cancer. Many studies have demonstrated that high Ki67 expression (with a cut-off value of 30% for the percentage of Ki67-expressing cells) is associated with poor prognosis and can predict anti-tumor therapy efficacy in breast cancer (Kurebayashi et al. 2014; Shao et al. 2021; Zhu et al. 2020). In melanoma, Ki67 expression has been proposed as a biomarker of metastasis and predictor of prognosis (Gimotty et al. 2005; Ladstein et al. 2010). However, the results of studies have not been consistent. As for AM, Wang et al. summarized and analyzed the characteristics and prognostic factors of 211 AM patients in China, and the results showed that Ki67 index in a continuous format was independently associated with prognosis (Wang et al. 2021). However, to the best of our knowledge, there are no additional studies confirming the predictive value of Ki67 in AM and no prognostic models have been previously established based on Ki67 expression for AM. Hence, this study aimed to summarize the clinicopathological and survival features across different Ki67 levels in AM patients and generate a predictive model to predict the survival of patients with AM.

## Methods

### Patients

The patients were identified from a cohort of 5758 patients with melanoma admitted to Peking University Cancer Hospital between 2006 and 2018 (Fig. S1). Eligibility criteria for enrollment were 1. Pathological diagnosis of AM (stage I, II, III or IV; based on the AJCC 8th cutaneous melanoma staging system); 2. Diagnosis data dated between 1 January 2006 and 31 December 2018; 3. Documented Ki67 expression identified by immunohistochemical staining. Data extracted from medical records included age, sex, primary site (sole vs. palm vs. nailbed), Breslow thickness (mm), ulceration (present vs. absent), initial date of diagnosis, clinical stage at diagnosis, LDH level, gene mutational status (BRAF, c-KIT, NRAS: mutated vs. wild-type), and date of death or date last known alive. The study was approved by the Medical Ethics Committee of Peking University Cancer Hospital.

### Immunohistochemistry

Formalin-fixed, paraffin-embedded samples retrieved from patients’ primary sites were cut into 5-μm sections. Immunohistochemical staining for Ki67 was performed using the immunoglobulin G1 mouse-antihuman Ki67 monoclonal antibody (clone MIB-1; DAKO, Carpenteria, CA, USA) and identified using an automated immunohistochemical staining system (DAKO Cytomation). Ki67 expression was assessed by two pathologists without knowledge of patients’ clinical data by calculating the proportion of positive melanoma cells per 100 cells. If their opinions differed, they would discuss the differences carefully to reach an agreement.

### Statistical analysis

According to previous studies, we defined patients in the Ki67 high or low group based on the percentage of cells expressing Ki67 ≥ 30% or less than 30%, respectively. Melanoma-specific survival (MSS) was defined as the time from the initial diagnosis to the date of melanoma-specific death. Pearson’s χ2 statistic or Fisher’s exact test was used to identify differences between groups. The Kaplan–Meier method was used to plot the survival curves and differences in MSS between groups were identified by log-rank test. A two-sided *P* value < 0.05 was considered statistically significant. All statistical analyses were performed using R version 4.2.2.

Univariate and multivariate Cox proportional hazards regression models were used to estimate hazard ratios (HRs) of risk factors for MSS. The factors related to prognosis from the results of univariate analysis were selected to establish the survival model. The patients were randomly divided into a training cohort (*n* = 219) and a validation cohort (*n* = 95). Then we analyzed the selected variables by Cox regression model and generated a nomogram in the training cohort. The index of concordance (C-index) and the area under the curve (AUC) were used to test the predictive potential of the model. A calibration plot was subsequently utilized to evaluate the consistency between the risk predictions generated by the model and the actual results. Net reclassification improvement (NRI) and integrated discrimination improvement (IDI) were used to estimate improvements in the predictive accuracy of the nomogram.

## Results

### Patient characteristics

Between January 2006 and December 2018, a total of 314 AM patients were finally enrolled in this study. We summarized the characteristics of all patients based on Ki67 ≥ 30% and Ki67 < 30% in Table 1. The ratio of male to female was 1.17:1 (169 vs. 145). The median age of all patients was 56.0 years. Ki67 < 30% was observed in 50.6% (159/314) AM patients and Ki67 ≥ 30% was observed in 49.4% (155/314) patients. Compared with patients with Ki67-low melanoma, the patients with Ki67-high melanoma were more likely to present with ulceration, thicker primary lesions, more lymph node metastases, distant metastasis and more advanced stages. The primary sites of all patients were soles (72.0%), nailbed (20.1%) and palm (8.0%), and the distribution was similar between patients with Ki67 ≥ 30% and Ki67 < 30% (*P* = 0.546). Hotspot mutational analysis was performed in 233 patients, and 11.1% (35/314) of patients were detected with the BRAF mutation, 4.8% (15/314) with the c-KIT mutation, and 12.1% (38/314) with the NRAS mutation. Elevated LDH levels were confirmed in 11.5% (36/314) patients. Of all patients, 87.6% (275/314) received complete resection and 68.8% (216/314) received interferon adjuvant therapy.

**Table 1:** Basic characteristics of AM patients

Characteristics	Ki67 < 30%	Ki67 ≥ 30%	Total	*P*-value
*N* (%)	159	155	314	
*Age*				
Mean (SD)	54.1(12.6)	55.3 (12.8)	54.7 (12.7)	0.400
Median (IQR)	56.0 (45.0–62.5)	56.0 (48.0–65.0)	56.0 (46.0 – 64.0)	
< 65	129 (81.1)	115 (74.2)	244 (77.7)	0.180
≥ 65	30 (18.9)	40 (25.8)	70 (22.3)	
*Sex*				
Male	82 (51.6)	87 (56.1)	169 (53.8)	0.486
Female	77 (48.4)	68 (43.9)	145 (46.2)	
*Stage*				
I	27 (17.0)	16 (10.3)	43 (13.7)	< 0.001
II	79 (49.7)	50 (32.3)	129 (41.1)	
III	39 (24.5)	62 (40.0)	101 (32.1)	
IV	14 (8.8)	27 (17.4)	41 (13.1)	
*Breslow thickness*				
≤ 1 mm (T1)	17 (10.7)	7 (4.5)	24 (7.6)	0.013
> 1–2 mm (T2)	33 (20.8)	29 (18.7)	62 (19.7)	
> 2–4 mm (T3)	49 (30.8)	35 (22.6)	84 (26.8)	
> 4 mm (T4)	60 (37.7)	84 (54.2)	144 (45.9)	
*Ulceration*				
Present	89 (56.0)	113 (72.9)	202 (64.3)	0.003
Absent	70 (44.0)	42 (27.1)	112 (35.7)	
*Lymph node metastasis*				
0	111 (69.8)	75 (48.4)	186 (59.2)	0.001
≥ 1–3	26 (16.4)	46 (29.7)	72 (22.9)	
≥ 4	22 (13.8)	34 (21.9)	56 (17.8)	
*Distant metastasis*				
No (M0)	145 (91.2)	128 (82.6)	273 (86.9)	0.036
Yes (M1)	14 (8.8)	27 (17.4)	41 (13.1)	
*Primary site*				
Sole	118 (74.2)	108 (69.7)	226 (72.0)	0.546
Palm	13 (8.2)	12 (7.7)	25 (8.0)	
Nailbed	28 (17.6)	35 (22.6)	63 (20.1)	
*Mutation*				
Wild type	79 (49.7)	66 (42.6)	145 (46.2)	0.302
BRAF	16 (10.1)	19 (12.3)	35 (11.1)	
c-KIT	4 (2.5)	11 (7.1)	15 (4.8)	
NRAS	18 (11.3)	20 (12.9)	38 (12.1)	
Missing	42 (26.4)	39 (25.2)	81 (25.8)	
*LDH*				
≤ 1 ULN	147 (92.5)	131 (84.5)	278 (88.5)	0.042
> 1 ULN	12 (7.5)	24 (15.5)	36 (11.5)	
*Surgery*				0.072
No	14 (8.8)	25 (16.1)	39 (12.4)	
Yes	145 (91.2)	130 (83.9)	275 (87.6)	
*Adjuvant interferon*				0.784
No	48 (22.6)	50 (32.3)	65 (31.2)	
Yes	111 (69.8)	105 (67.7)	216 (68.8)	

### Ki67 and survival

**Fig. 1:**
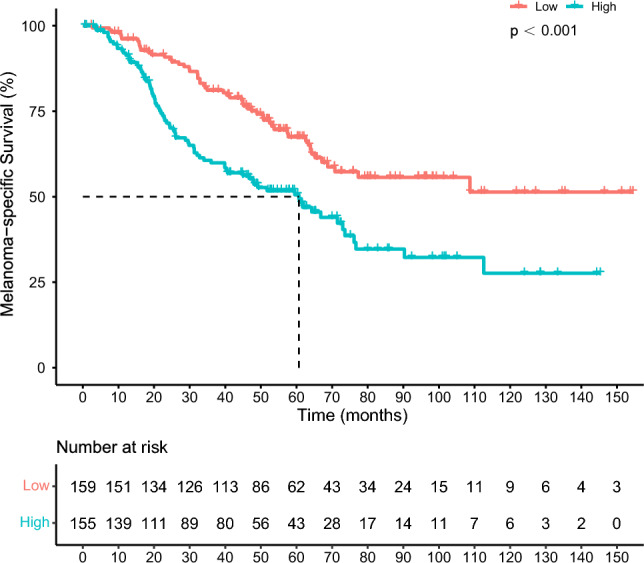
Kaplan–Meier curves of melanoma-specific survival according to different Ki67 levels

By July 19, 2022, at a median follow-up time of 62.9 months, the median MSS for all patients was 71.5 months (95% confidence interval [CI] 63.9–113.0) and the 1-, 3-, 5- and 8-year survival rates were 94.1% (95% CI 91.5–96.8), 71.1% (95% CI 66.1–76.5), 59.2% (95% CI 53.5–65.5) and 44.5% (95% CI 37.7–52.4), respectively. Patients in the Ki67-high group had a lower median MSS than those in the Ki67-low group (60.7 months vs. not reached, *p* < 0.001; Fig. 1). The 1-year MSS rates were similar between the two groups (92.0% vs. 96.1%). However, the MSS rates at 3, 5, and 8 years were lower for patients in the Ki67-high group (60.6%, 50.6% and 32.2%, respectively) than for those in the Ki67-low group (81.1%, 67.5% and 55.7%, respectively).

To further elucidate the prognostic value of Ki67 on MSS, we conducted univariate and multivariate Cox proportional hazards regression analyses (Table 2). In univariate analysis, high Ki67 expression, male, Breslow thickness > 4 mm, ulceration, lymph node metastasis, distant metastasis, sole melanoma, elevated LDH levels and complete surgical resection were associated with worse survival outcomes. The final multivariate regression model indicated that Ki67, lymph node metastasis and primary site were independent prognostic factors for MSS. Compared with sole melanoma, nailbed melanoma was a relatively protective prognostic factor [HR: 0.59 (95% CI 0.36 – 0.98), *p* = 0.040]. The adjusted HR for lymph node metastasis (≥ 4 vs. 0) was 3.00 (95% CI 1.94 – 4.66, *p* < 0.001), which was higher than the adjusted HR of 1.58 for Ki67 expression (95% CI 1.08 – 2.30, *p* = 0.017). We also examined Ki67 level in a continuous format for multivariate Cox regression, and results showed that Ki67 level was an independent factor for MSS (HR: 1.02 [95% CI 1.01 – 1.03], *p* < 0.001).

**Table 2:** Univariate and multivariate Cox regression analysis results for prognostic factors affecting MSS

Groups	Univariate		Multivariate	
	HR (95% CI)	*P*	HR (95% CI)	*P*
*Ki67*				
≥ 30% vs. < 30%	1.97 (1.39–2.78)	< 0.001	1.58 (1.08–2.30)	0.017
*Age*				
≥ 65 vs. < 65	1.42 (0.97–2.08)	0.068		
*Sex*				
Male vs. Female	1.71 (1.21–2.42)	0.003	1.40 (0.97–2.01)	0.071
*Breslow thickness*				
> 1–2 vs. ≤ 1	1.97 (0.66–5.81)	0.222	1.10 (0.36–3.34)	0.861
> 2–4 vs. ≤ 1	3.38 (1.19–9.56)	0.022	1.68 (0.57–4.93)	0.348
> 4 vs. ≤ 1	5.75 (2.10–15.78)	0.001	2.53 (0.88–7.28)	0.085
*Ulceration*				
Present vs. Absent	1.98 (1.35–2.91)	0.009	1.33 (0.88–2.03)	0.180
*Lymph node metastasis*				
≥ 1–3 vs. 0	2.04 (1.35–3.07)	0.001	1.47 (0.94–2.30)	0.093
≥ 4 vs. 0	4.33 (2.85–6.56)	< 0.001	3.00 (1.94–4.66)	< 0.001
*Distant metastasis*				
Yes vs. No	3.58 (2.39–5.36)	< 0.001	1.62 (0.60–4.39)	0.341
*Primary site*				
Nailbed vs. Sole	0.59 (0.36–0.95)	0.030	0.59 (0.36–0.98)	0.040
Palm vs. Sole	0.67 (0.35–1.28)	0.228	0.60 (0.30–1.20)	0.146
*Mutation*				
BRAF vs. Wild	0.77 (0.42–1.42)	0.401		
c-KIT vs. Wild	1.29 (0.62–2.69)	0.497		
NRAS vs. Wild	1.33 (0.82–2.16)	0.241		
*LDH*				
> 1 ULN vs. ≤ 1 ULN	1.78 (1.09–2.90)	0.021	1.13 (0.68–1.90)	0.636

### Predictive model

**Fig. 2:**
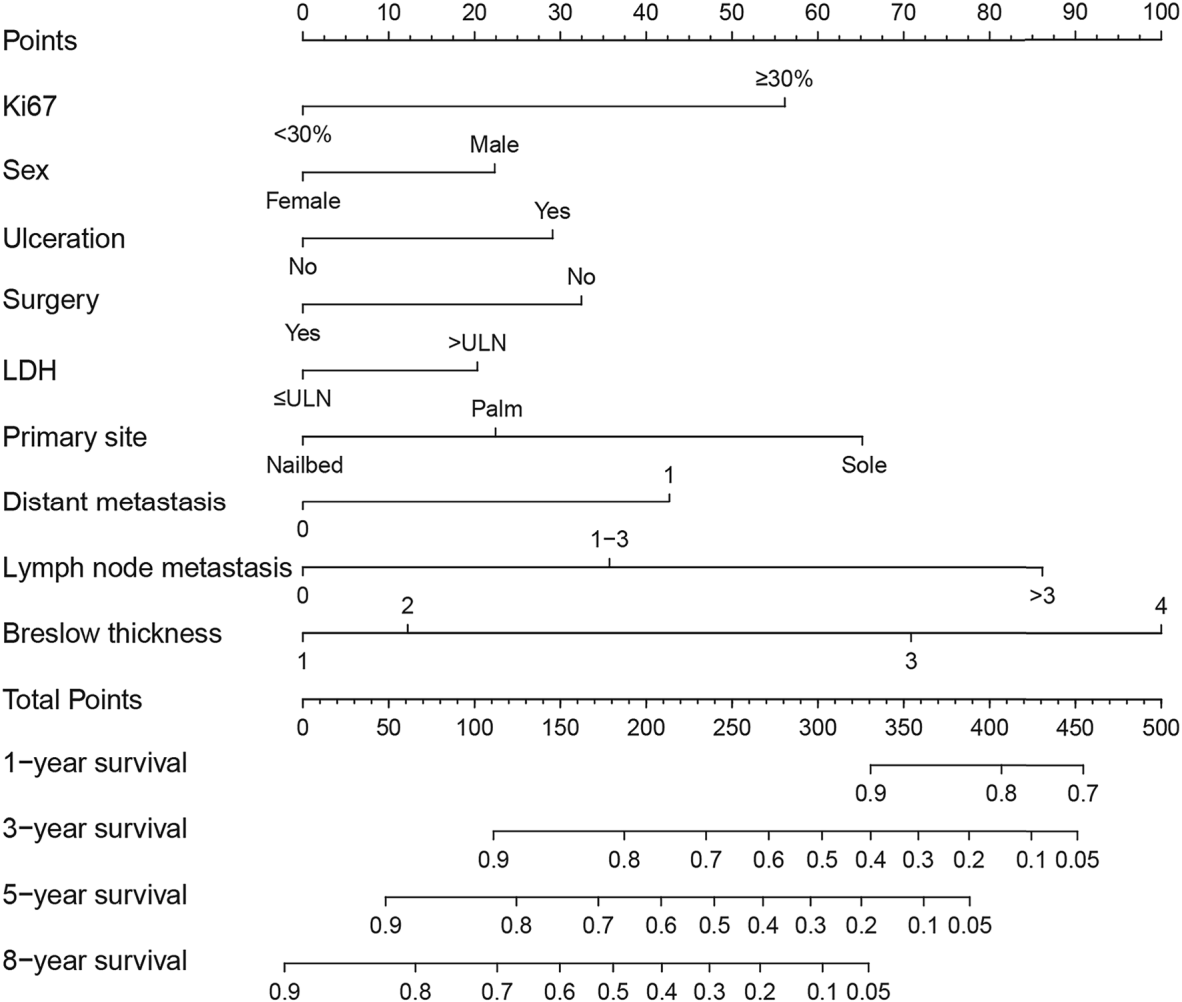
Nomogram of acral melanoma

On the basis of the results of univariate Cox regression analysis, a number of baseline parameters were selected to establish a clinical model, including Ki67 expression, sex, Breslow thickness, ulceration, lymph node metastasis, distant metastasis, primary site, LDH level and complete surgical resection. A nomogram (Fig. 2) was constructed on the basis of multivariate analysis of selected characteristics in the training cohort. Each predictive factor corresponds to the score at the top of the nomogram, and the sum of the scores for each patient corresponds to the 1-, 3-, 5- and 8-year MSS rate at the bottom. As can be seen from the nomogram, Breslow thickness, lymph node metastasis, primary site and Ki67 expression had the greatest impact on MSS in patients with AM.

**Fig. 3:**
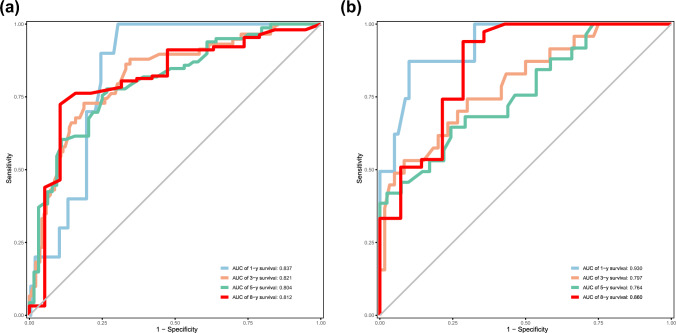
Receiver operating characteristic curves for the nomogram in the training cohort (**a**) and validation cohort (**b**)

**Fig. 4:**
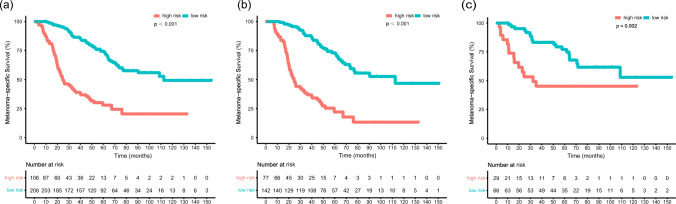
Kaplan–Meier curves of melanoma-specific survival for patients in low- and high-risk groups. **a** All patients included in the study. **b** Patients in the training cohort. **c** Patients in the validation cohort

Then we evaluated the performance of the model with several validation parameters. The C-index was 0.765 in the training cohort and 0.758 in the validation cohort, which indicated good accuracy of the predicting model. Additionally, the AUC values were both high in the training and validation cohorts (Fig. 3). The calibration plots demonstrated good agreement between the predicted and actual MSS probabilities (Fig. S2). Moreover, compared with the predicting model of the AJCC staging system alone, the NRI values at 1, 3, 5 and 8 years were 0.118, 0.357, 0.377 and 0.317, while the IDI rates at 1, 3, 5 and 8 years were 0.005, 0.100, 0.128 and 0.123, respectively, suggesting superior predictive performance of our new model. The patients in this study, as well as those in the training and validation sets were respectively divided into low-risk and high-risk groups by the optimal cut-off value calculated from the nomogram, and the survival curves of the two risk groups were generated separately (Fig. 4).

## Discussion

AM, the most common melanoma subtype in Asia and Africa, is characterized by poor survival due to its more advanced stage at diagnosis as well as negative prognostic and genetic features (Carrera and Puig-Butille 2018; Lv et al. 2016). A series of publications have reported various prognostic markers for melanoma survival, but the results are controversial among those studies. The proliferation marker Ki67 has been discovered to be linked to survival of many malignancies, including melanoma. However, the prognostic status of Ki67 for patients with AM is less known. In this regard, we analyzed in this study the relationship between Ki67 and the clinicopathological and survival profiles of patients with AM and developed a nomogram prediction model to predict MSS of patients with AM at 1, 3, 5, and 8 years. To the best of our knowledge, this study has used the largest sample size to elucidate the relationship between Ki67 and AM and is the first study to build a model incorporating Ki67 for predicting survival of AM in a Chinese population.

Ki67 as a biomarker reflecting cell proliferation rate has been explored in predicting chemotherapy response and prognosis in various tumors. A series of studies have demonstrated that high Ki67 expression is correlated with poor prognosis in prostate, lung, serous ovarian, clear-cell renal cell and pancreatic cancers (Adams et al. 2009; Gayed et al. 2014; Genç et al. 2018; Lei et al. 2013; Pascale et al. 2016,). In particular, Ki67 expression has been used as an index to classify patients with breast cancer into different risk categories and to guide adjuvant or neoadjuvant therapy (Aleskandarany et al. 2011; Chen et al. 2018; Petrelli et al. 2015). Additionally, a series of studies found a correlation between Ki67 and patients with melanoma. Ki67 is known to be increased in melanoma and not in nevi, which could help pathologists to distinguish melanoma from nevus in challenging cases (Nielsen et al. 2012; Torres-Cabala et al. 2020). In cutaneous melanoma, overexpression of Ki67 was demonstrated to correlate with tumor grade, metastasis, melanoma-specific mortality, disease-free survival and overall survival (Gimotty et al. 2005; Nielsen et al. 2013; Väisänen et al. 2011). In mucosal melanoma, a few studies have indicated that Ki67 is a predictive indicator for both survival and adjuvant chemotherapy (Ben-Izhak et al. 2002; Kim et al. 2008; Tang et al. 2022). However, scanty information about the relationship between Ki67 and AM is available. There has only been one retrospective study depicting the prognostic value of Ki67 in AM patients. From analysis of a single-center series of 211 AM patients, Wang et al. found that the Ki67 index in a continuous format was independently associated with survival (Wang et al. 2021). In the present study, we enrolled 314 AM patients and summarized their clinicopathologic characteristics. The results suggested that Ki67 index in a continuous format correlated with MSS, which is consistent with the findings of Wang et al. In addition, we divided patients into two groups based on a cut-off Ki67 value of 30% and observed significantly favorable MSS for patients with Ki67 < 30% compared to patients with Ki67 ≥ 30%. In multivariate analysis, high Ki67 expression was an independent indicator of poor MSS in AM patients, which provided evidence that Ki67 expression could be used as a stratification factor to predict survival in AM patients in the future. In breast cancer, patients with high Ki67 expression (> 30%) had worse disease-free survival and overall survival after adjuvant tamoxifen therapy (Elzawahry et al. 2013), while they could benefit from chemotherapy (Criscitiello et al. 2014). Interestingly, Tang et al. discovered that for patients with Ki67 ≥ 30%, relapse-free survival was longer with temozolomide-based adjuvant chemotherapy compared to high-dose interferon-α2b adjuvant therapy (Tang et al. 2022). Whether Ki67 can play a similar role in AM warrants further investigation.

Among 314 AM patients, 49.4% of patients showed Ki67 ≥ 30% at diagnosis in our study. With respect to Breslow thickness, the percentage of patients with thickness > 4 mm was higher in patients with high Ki67 expression than in the low Ki67 group, and this was comparable to another study in which tumors with high Ki67 expression (> 10%) were thicker than those with low Ki67 expression (Väisänen et al. 2011). With regard to ulceration, tumors with low Ki67 were less likely to appear with ulceration. Furthermore, patients in the Ki67-high group tended to present at a later stage, with more lymph node metastases and distant metastasis. Ki67 expression was significantly lower in patients with nonmetastatic disease (stage I/II) than in those with metastatic disease (stage III/IV), but there was no difference between either stage I vs. II or stage III vs. IV. Other characteristics such as age, sex, primary site and mutation status were found to be unrelated to Ki67. Many publications have reported that age, Breslow thickness, ulceration status, stage, primary site and LDH correlated to prognosis in AM patients (Teramoto et al. 2018; Wang et al. 2021; Wei et al. 2020; Wei et al. 2022a, b). However, the results have been conflicting for some variables. In our study, age, Breslow thickness, ulceration status, LDH and mutation status were not prognostic factors for MSS, whereas lymph node metastasis, primary site and Ki67 were independently associated with MSS. Wei et al. discovered the prognosis for sole melanoma was worse than for palm and nailbed subtypes (Wei et al. 2020), which is consistent with our findings. Ki67 expression in a continuous format was found to be an independent factor for MSS in our analyses, which was in alignment with the findings of Wang et al. (Wang et al. 2021). As a grouping variable, Ki67 was still independently correlated with MSS.

In this study, a nomogram prediction model incorporating Ki67 with other clinical parameters was constructed to predict 1-, 3-, 5-, and 8-year MSS rates in patients with AM. The impact of Ki67 expression on MSS was only surpassed by Breslow thickness, lymph node metastasis and primary site in this nomogram. The C-index values of the nomogram were both favorable in the training and validation cohorts. The AUC reached above 0.8 at the observed timepoints in the training cohort, and reached approximately 0.8 in the validation cohort. The nomogram achieved higher accuracy than that of AJCC staging system, as the NRI was above 0. The IDI showed that the predictive ability for 1-, 3-, 5- and 8-year survival was 0.5%, 10.0%, 12.8%, and 12.3%, respectively, compared with that of AJCC staging system.

Our established nomogram has certain advantages, especially in its practicality. First of all, the nomogram has integrated different prognostic and decisive variables that are easy to obtain and evaluate, which means it can help clinicians make an early assessment of the prognosis of patients with AM quickly and conveniently. In addition, the visual nomogram can enhance patients’ understanding, promote communication between doctors and patients, and improve patients’ cooperation. Furthermore, clinicians can identify patients with low overall survival earlier using this model, and then undertake more active and individualized treatments to improve the overall survival of AM patients.

There were some limitations in our study. First, some potential selection bias and information bias may exist as a result of the respective nature of the study. Secondly, the survival data may be affected by disease evolution and subsequent treatment; thus some accuracy of the nomogram might be sacrificed. Thirdly, this was a single-center study consisting of limited samples and we did not perform external validation for the nomogram. Therefore, the predictive model warrants further validation in a larger cohort, and a prospective study is required to elucidate the prognostic value of Ki67 for AM.

In this study, we described Ki67 expression in AM patients and analyzed its association with other clinicopathologic characteristics of AM patients. The results demonstrated that Ki67 as a stratification index can serve as an independent prognostic factor for AM patients. Furthermore, we have established the first nomogram incorporating Ki67 for AM patients based on a Chinese population, and the nomogram achieved better predictive power than the traditional AJCC staging system. The nomogram may thus help clinicians predict survival probability and guide individualized clinical therapy in AM patients in the future.

### Supplementary Information

Below is the link to the electronic supplementary material.

Supplementary file1 (DOCX 374 KB)

## Data Availability

The data presented in this study are available upon reasonable request from the corresponding author.
