# Supplementary material for: A nomogram incorporating Ki67 to predict survival of acral melanoma
Source: J Cancer Res Clin Oncol. 2023 Jul 20;149(14):13077–85. doi: 10.1007/s00432-023-05127-w (PMC10587210; doi:10.1007/s00432-023-05127-w)

Fig. S1 Flowchart of inclusion and exclusion criteria used in this study


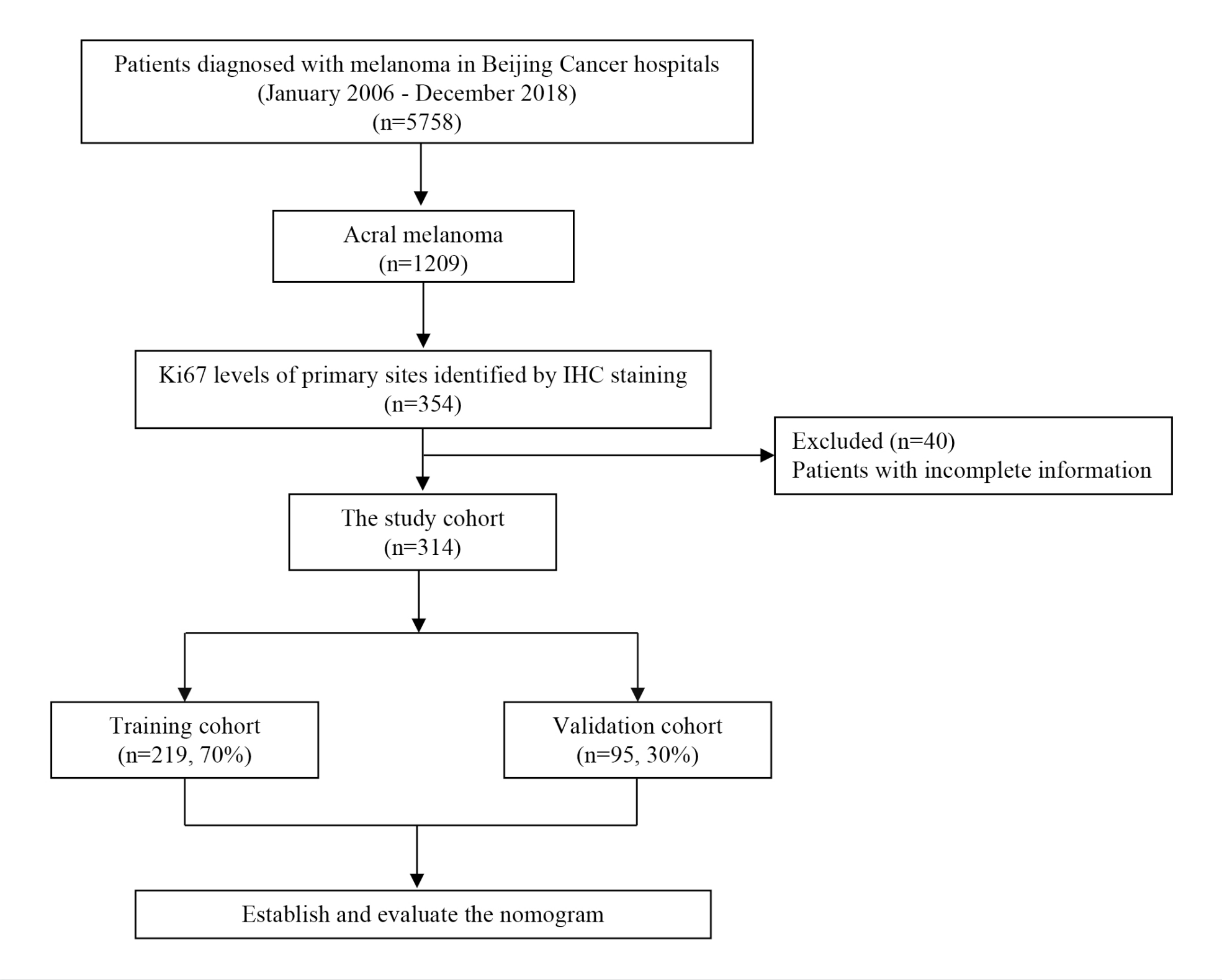


Fig. S2 Calibration curves of the nomogram for predicting overall survival at 1-year, 3-years, 5-years and 8-years in the training cohort (a, c, e, g) and validation cohort (b, d, f, h)


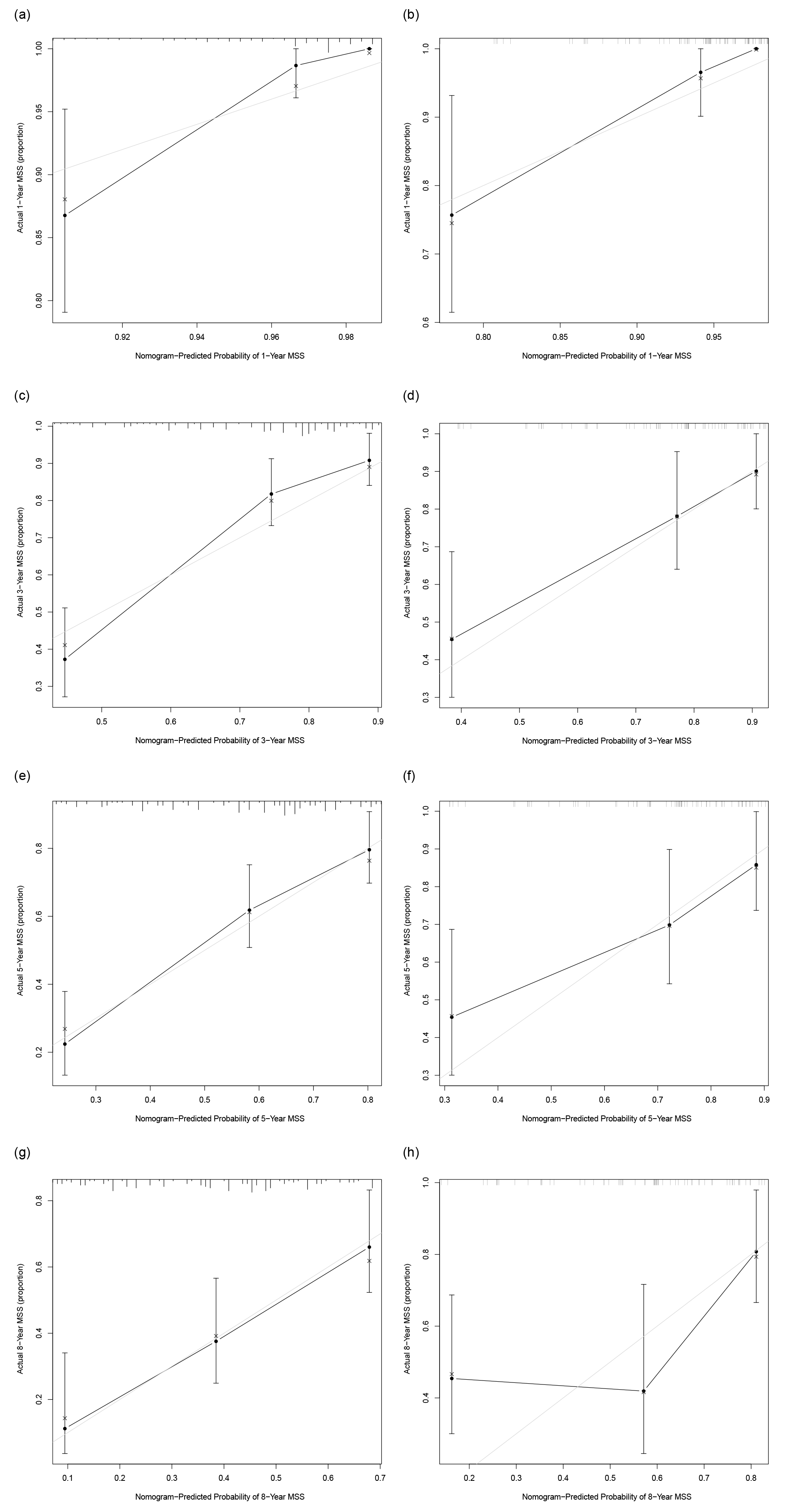

Supplement: Supplementary file 1 — Supplementary file1 (DOCX 374 KB) [file 432_2023_5127_MOESM1_ESM.docx]
